# Emergence of consensus as a modular-to-nested transition in communication dynamics

**DOI:** 10.1038/srep41673

**Published:** 2017-01-30

**Authors:** Javier Borge-Holthoefer, Raquel A. Baños, Carlos Gracia-Lázaro, Yamir Moreno

**Affiliations:** 1 Qatar Computing Research Institute, HBKU, Doha, Qatar; 2 Internet Interdisciplinary Institute (IN3), Universitat Oberta de Catalunya (UOC), Barcelona, Spain; 3 Institute for Biocomputation and Physics of Complex Systems (BIFI), Universidad de Zaragoza, 50018 Zaragoza, Spain; 4 Department of Theoretical Physics, Faculty of Sciences, Universidad de Zaragoza, Zaragoza 50009, Spain; 5 Institute for Scientific Interchange (ISI), Torino, Italy

## Abstract

Online social networks have transformed the way in which humans communicate and interact, leading to a new information ecosystem where people send and receive information through multiple channels, including traditional communication media. Despite many attempts to characterize the structure and dynamics of these techno-social systems, little is known about fundamental aspects such as how collective attention arises and what determines the information life-cycle. Current approaches to these problems either focus on human temporal dynamics or on semiotic dynamics. In addition, as recently shown, information ecosystems are highly competitive, with humans *and* memes striving for scarce resources –visibility and attention, respectively. Inspired by similar problems in ecology, here we develop a methodology that allows to cast all the previous aspects into a compact framework and to characterize, using microblogging data, information-driven systems as mutualistic networks. Our results show that collective attention around a topic is reached when the user-meme network self-adapts from a modular to a nested structure, which ultimately allows minimizing competition and attaining consensus. Beyond a sociological interpretation, we explore such resemblance to natural mutualistic communities *via* well-known dynamics of ecological systems.

Nowadays, online social networks constitute mainstream ways to communicate, exchange opinions, and reach consensus^1^,^2^,^3^,^4^. They are characterized by a multichannel information flow and by an adaptive topology. In recent years, it has increasingly become evident that competition significantly shapes the topology of and the dynamics on these information-driven platforms^5^,^6^, also at the *macro* scale^7^. Given the many sources of information to which a typical individual is exposed, it is likely that the economy of attention rules the system dynamics^8^: even opinion-aligned individuals compete to increase their visibility among other peers, given the limitations of our social brain^5^,^6^. Such competition may not be direct, but rather mediated by the symbols (memes) that take part in the communicative interaction^9^,^10^ –which similarly compete^11^ for the attention of those who produce and consume them (users).

The accent on *intra-class* (user-user, meme-meme) competition renders however a partial picture. Turning to *inter-class* interactions, these appear under the form of mutualism: the choice of more frequent memes increases the visibility of individuals, which makes the popularity of those memes even larger, thus decreasing the likelihood that other competing memes also become fashionable. Under this diversity of actors and connections, an information-driven system can be thought of as a bipartite network in which individuals and memes concurrently compete (within their class) and cooperate (between classes), see Fig. 1. Such a system is reminiscent of those that have been reported in other areas, be them plant-animal^12^,^13^,^14^ or manufacturer-contractor networks^15^, in which nestedness –a widely reported structural pattern in mutualistic ecological systems– is a prominent topological feature. The question is then whether similarities at the dynamical level (same type of interactions) are mirrored at the structural one, and (if so) why a nested architecture, in which specialists –interacting with only a few partners– tend to be connected with generalists –those interacting with many others, emerges.

Microblogging platforms stand out as the perfect test bed to answer such question, since messages are explicitly limited to a small number of characters –competition in such restricted environments is fierce, and the choice of memes (hashtags in Twitter, for instance) critically determines the success of the message (outreach) and its lifetime on the system (persistence). Moreover, even though the finding of a nested architecture in bipartite communication networks would be suggestive, online social networks additionally provide us with time-resolved data, which makes it possible to trace back the origins of the nested pattern –at variance with all previous works: we have scarce evidence of how nestedness arises in nature, given the observational limitations and costs of fieldwork^16^. This is the reason why ecologists have focused rather on other aspects^17^,^18^,^19^, letting aside the temporal dimension, i.e., the growth and evolution of the system and the emergence of nested patterns.

Here we show that in the information ecology context, it is possible to monitor the emergence of a nested architecture out of an incipient system, which, surprisingly enough first appears under the dominant form of a modular network. To do so, we represent a communication platform as a bipartite graph where connections exist only between agents (users) and the symbols (memes) they produce. Exploiting the inherent time-stamped nature of the data, the bipartite setting yields longitudinal observation of initially modular-and-nested, then nested-only structures from large, public collections of online microblogging data. Additionally, we perform extensive numerical simulations on synthetic networks and find that the observed modular-to-nested transition is due to the fact that the user-meme community is pushed towards a nested architecture to accommodate mutualistic interactions –as opposed to antagonistic ones intrinsic to a modular scenario^20^,^21^,^22^. Our results provide a novel mechanism to explain the emergence of consensus in social systems, and clear the path for a new set of concepts and tools –borrowed from ecology– to be applied in such systems. Last, but not least, our observation of an *empirical* modular-to-nested structural transition can shed light into the problem on the origin of nested architectures, which remains an elusive question.

## Results

**Figure 1:**
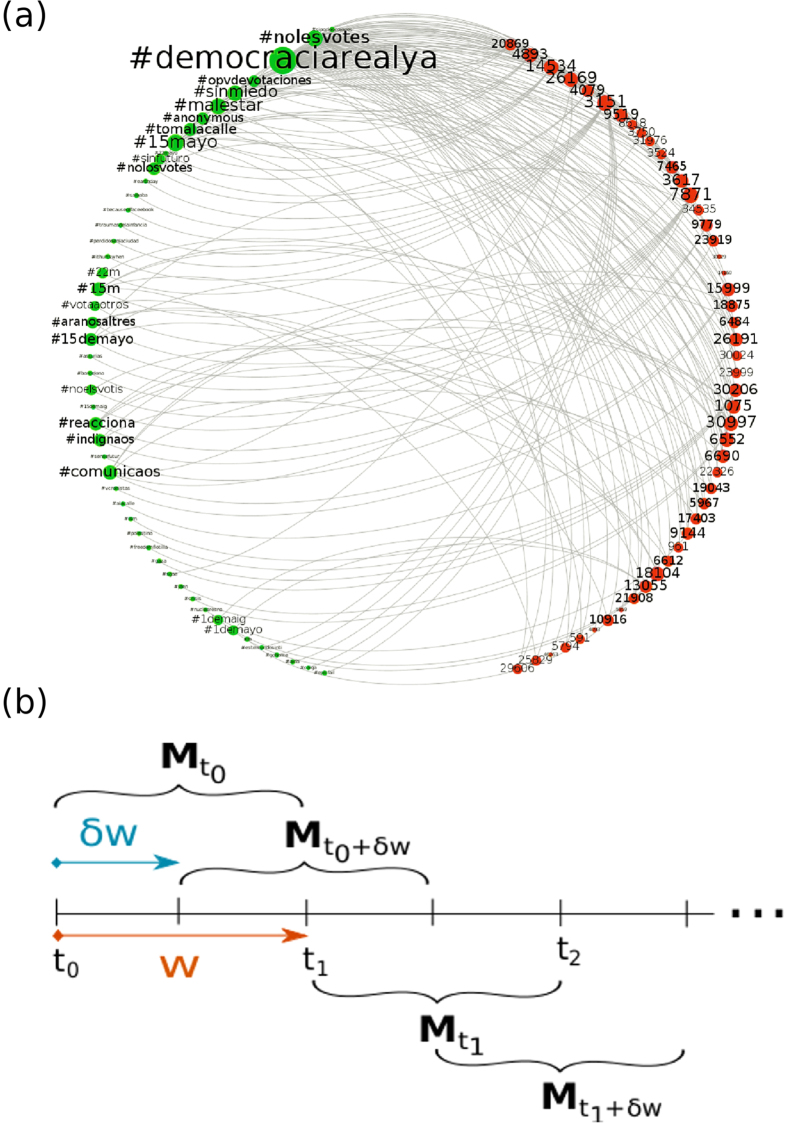
Time-dependent Mutualistic Networks. Bipartite representation of the user-hashtag interaction network at the beginning of the observation period (**a**). An undirected link is shown whenever a user (represented here by an integer number) authors a tweet containing the corresponding hashtag. The size of hashtags and users is proportional to their frequency/activity. Panel (b) sketches the sliding-window scheme, which produces the matrices **M**_*t*_ that contain the interactions between users and hashtags starting at time *t*_*0*_ and lasting till time *t*, with *w* = *t* – *t*_*0*_.

We first present results for a dataset corresponding to civil protests in Spain (15 M movement) that resonated on Twitter, in the period April-May 2011^23^,^24^. The dataset was obtained from a predefined set of keywords relevant to the movement (section A and Appendix of the Supplementary Materials (*SM*) describe in depth all datasets used in the work). These data, taken in *w*-wide sliding windows, contain all the necessary information to build time-resolved bipartite networks –*who* said *what*, and *when*– suitably encoded as a rectangular, time-dependent matrix. Specifically, the Twitter stream is parsed and bipartite graphs –see Fig. 1a and 1b– are built up as follows: first, time windows are set to a fixed, arbitrary, *w* = *t*_*2*_– *t*_*1*_ width. We then choose the *n* most active users and the *m* memes (hashtags) that those users produced within that time interval. This bipartite network is encoded in an *n* × *m* rectangular binary matrix, **M**_*t*_, where *t* indicates the origin of the time window *w* and *M*_*u,h*_ = 1 if user *u* mentioned the hashtag *h* within the period spanning from *t*_*1*_ to *t*_*2*_ and zero otherwise. This procedure allows generating bipartite networks as time goes on by using a rolling-window scheme to evaluate the evolution of the system, such that a window at time *t* has a *φw* overlap with that at time *t* – *w (φ* = 0.5 in the results reported here; for *φ* closer to 1.0 results com at higher resolution, whereas *φ* = 0.0 implies non-overlapping windows).

Once the networks associated to the 15 M social movement at different times are assembled, we proceed to analyze their structure focusing on two topological characteristics. As the interest is in inspecting whether groups of individuals using the same memes build up, we first look for the optimal modular partition of the nodes through a community detection analysis^25^,^26^, applying a simulated annealing heuristics to maximize Barber’s^26^ modularity *Q*.

Next, we study whether nested patterns arise in the system. Here we evaluate nestedness following the findings by Bell *et al*.^27^,^28^ and further developed in Staniczenko *et al*.^29^, who showed that it is given by the maximum eigenvalue of the *(n* + *m)* × *(n* + *m)* adjacency matrix of the network, i.e. the square matrix counterpart of **M**_*t*_. As shown in Fig. S2, our results are robust against other existing measures of nestedness (i.e., NODF^30^). For details on both *Q* and nestedness, see *Materials and Methods*, and Sections B and C in the *SM.*

**Figure 2:**
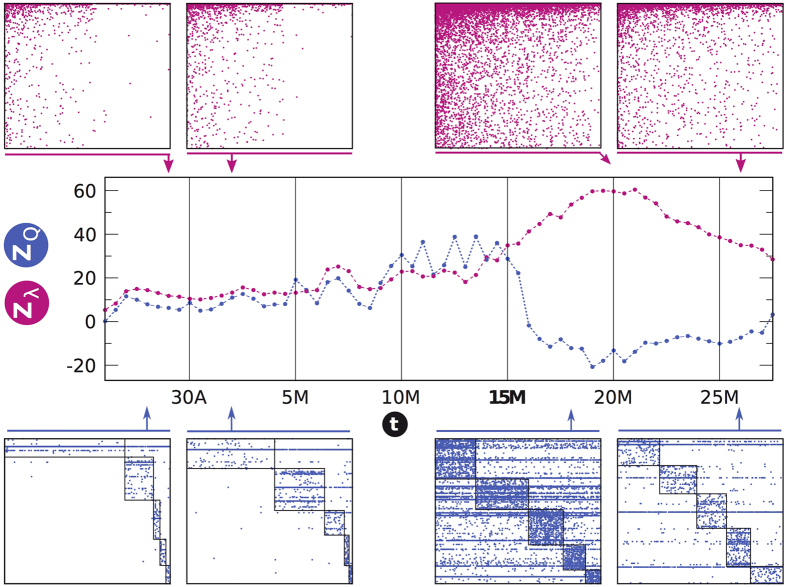
Modularity and nestedness bifurcate at the onset of system-wide attention. The central panel shows the evolution of modularity and nestedness, as standardised z-values. Remarkably, both metrics evolve in a coupled way up to the onset of the main protests (around May 15). At this point, modularity collapses, whereas nestedness continues growing towards its peak value coinciding with the political movement’s central dates –that of the largest demonstrations across the country (May 17–20th). Top panels represent four snapshots of the data –encoded as bipartite networks–, rows and columns are sorted in decreasing connectivity order (for an optimal visualization of nested patterns, if they exist). Similarly, lower panels represent the exact same matrices, where rows and columns are sorted module-wise (for an optimal visualization of the community structure).

Figure 2 shows the results of the application of these structural analyses for the 15 M dataset and a window width of *w* = 1 day. If we focus on the days around which the main demonstrations happened (May 15th and onwards), we see that the network presents a highly nested profile. This alone is a quite interesting result, as it implies that when the activity around certain topic peaks, the user-meme system is highly nested. Note that this scenario is more optimal for information diffusion than a predominantly modular topology, as in the latter architecture information flow can get stuck and never reach throughout the whole system. Thus, our findings contribute yet another example of commonalities between ecological, human^15^,^31^ and proto-cultural^32^ systems –for which we typically have static perspectives (but see ref. 33, 34, 35).

Importantly, we can trace back in time the emergence of the final nested state by inspecting the structure of the matrix **M**_*t*_ at different times *t*. With few exceptions, from the very the beginning of the observation time (April 25, 2011) the network exhibits significant (*z*_*Q*_ > 1.96) modularity and nestedness (*z*_*λ*_ > 1.96) values. This means that before the general onset of collective attention around the 15 M activity, the (proto-) topic is composed of a set of modules (Fig. 2, bottom left) which hardly interact with the rest of the system. At the same time, the structure of the network is nested (Fig. 2, top left). Both patterns exhibit a coupled growing trend (*r* = 0.7997) for some time, suggesting that discussion communities become clearer and more internally organized. This picture however changes as the movement gains momentum and consensus arises. Indeed, around the climax of the event (May 15–17) we observe an abrupt transition, i.e., nestedness keeps increasing as modularity collapses in a marked anti-correlated pattern (*r* = −0.7819). After such transition, the architecture of the network is radically different.

The compelling evidence of nested patterns provides a parsimonious explanation of how large amounts of activity can coexist with natural constraints to attention and memory. The user-meme network self-organizes towards a nested structure minimizing competition and facilitating the coexistence of individual participants^36^. Even when the network is predominantly modular, nestedness appears to have significant values well beyond random counterparts, which already indicates the existence of an incipient consensus around sub-topics. Moreover, the unraveled structural change to a highly nested-only architecture allows interpreting the evolution of the Spanish mobilization episodes as a build-up effort from segregation (scattered activists acting locally) to coordination (a global movement with a well-defined and shared main message).

**Figure 3:**
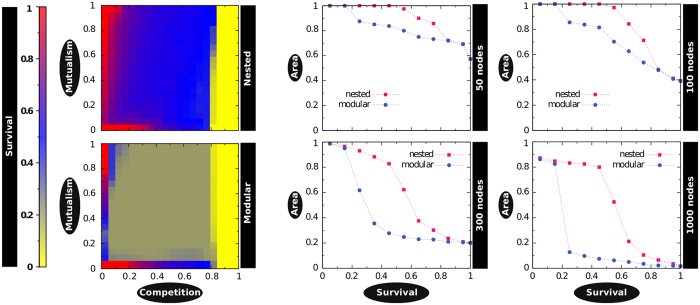
Modular and nested architectures under mutualistic dynamics. Two synthetic networks with the same size (*N* = 1000) and density (*ρ* = 0.25), but different architecture (modular, nested), exhibit radically different outcomes when the mutualistic dynamical framework is applied on them via extensive numerical simulations. Left: Persistence as a function of the competition *β* and mutualism *γ* terms. For a wide range of parameters the modular network shows poor survival; conversely, the nested architecture performs equally or better than the modular counterpart in any given region. Right: differences in the “survival areas” increase with size, which indicates that the pressure for an architectural shift (modular to nested) grows as new nodes (users and hashtags) join the system. Note that the *x*-axis in the right panels (“Persistence”) corresponds to the *z*-axis (color code) in the left panels. All results are averaged over 1000 realizations. Additional results for other sizes and densities can be found in Figs S7 to S11.

Such interpretation in sociological terms can be quantitatively supported if we actually explore the survival conditions under which the topic can persist. To do so, we build a set of synthetic networks that purposefully present an almost perfect modular architecture, and an almost perfect nested structure (see section E.1 in the *SM* for details), mimicking the initial and “climax” state of the real system, April 25–30 and May 15–20 respectively. To each pair (equal size and equal link density) of these networks, we apply the mutualistic dynamics proposed by Bastolla *et al*.^36^ exploring a wide range of model parameters’ values (see *Materials and Methods* and Section E.2 in *SM*). The aim is to compare the persistence of these two distinct topologies when equilibrium is reached. The first noticeable finding shows that the nested architecture presents large areas in the parameter space for which the system largely survives, whereas the modular structure does not (Fig. 3a). In all the cases (see additional results in Figs S7 to S11) it is possible (and actually very frequent) to observe high persistence for the nested architecture whereas it is low for the modular one, but never the other way around. In this context, the persistence is defined as the survival of a hashtag or user once the system has become stable, while the survival rate represents the final diversity (*i.e.*, number of users and hashtags in the steady state) relative to the initial collection. Then, the survival area represents the region with a survival rate greater than a given value (see Section E.2 of the *SM*). We systematically compare the survival areas for pairs of systems with different sizes and densities (Fig. 3b) and two remarkable facts stand out: first, nested architectures consistently out-survive modular ones. Second, the difference in survival areas increases with network size, being narrower for small system sizes. This latter finding suggests the reason why topic-centered bipartite networks in information systems exhibit a modular structure while they remain small-sized: the pressure for an architecture shift remains low, as the transition towards a nested topology does not yet present a critical advantage in terms of the survival of the topic. In other words, when a topic is emerging, and thus its user-meme network is small, it needs to reach a critical mass (here the size) and self-adapt to a nested architecture to increase the likelihood of topic’s survival.

**Figure 4:**
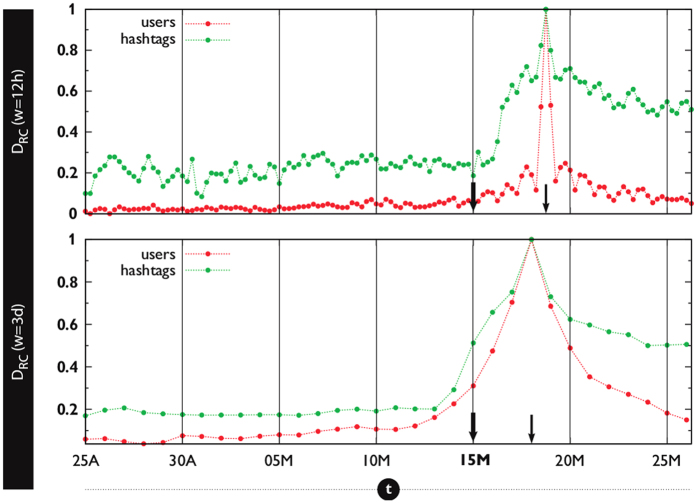
Topical consistence over time despite user turnover. We track the similarity *D*_*RC*_ of the generalist cores of the network in time (*C*_*t*_) with respect to a fixed reference (the core of the network when the maximum of the nestedness is observed, *C*_*max*_). Results for different *w* (12 h in the top panel; 3 days in the lower) show that only hashtags build a stable core, guaranteeing the semantic coherence of the topic across time; whereas the core of users suffers a high rate of turnover, indicating that users are frequently pushed to and from the periphery of the network.

It is possible to get further insights into the microscopic mechanisms behind the modular-to-nested topological transition. As seen from Fig. 2, once the nested patterns begin to dominate the network structure –around the day when the movement fully develops–, nestedness remains at high levels for some time. This makes it possible to consistently track the set of users and memes that accumulate many interactions (generalists) and inspect whether these sets are time-independent. To this end, we identify which nodes and which memes assemble the core^37^,^38^ of the network at different times. The core can be thought of as the set of most generalist nodes (users and memes) in the network, see section F of the *SM* for further details. Figure 4 compares the resemblance to the “reference core” *D*_*RC*_, i.e. similarity between a snapshot’s core (*C*_*t*_) and the one extracted when the nestedness is maximal (*C*_*max*_) (see section F in the *SM* for a definition). Notably, for both *w* = 12 h (top panel) and *w* = 3 days (bottom) there is a high turnover in users who occupy the core: in most snapshots *t*, only 0–10% of the users in *C*_*max*_ are also present in *C*_*t*_, even when the network’s architecture has reached the nested stage. Instead, hashtags have a much more stable core –around 20% of the *C*_*max*_ is shared during the entire observation window, and values above 50% are reached after the movement onset and beyond. These results suggest that it is the set of generalist memes, rather than the existence of generalist individuals, that takes the burden of the topic’s persistence in time. Indeed, it is less costly to linger on a set of hashtags –the *passive* elements of the system^39^– as they are not subject to users’ limitations (sleep, attention focus, etc.), with high volatility of new users who enter and leave the core rather intermittently. As shown in Fig. S4, these results are robust to different window widths.

**Figure 5:**
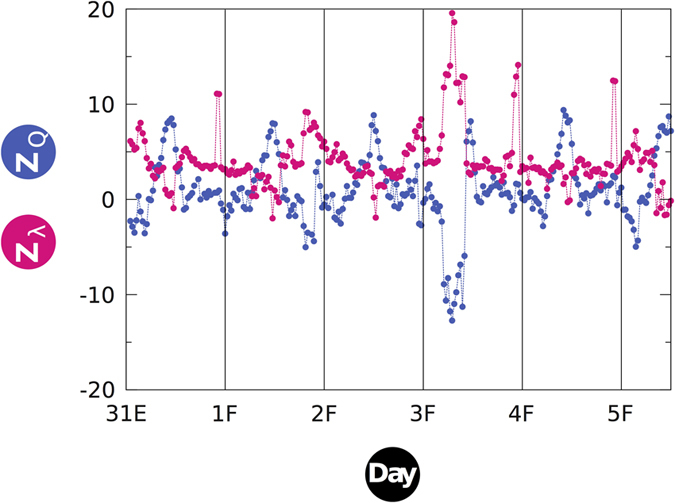
Maximum nestedness marks ephemeral topics at faster time-scales. Unfiltered, topic-independent Twitter traffic offers similar evidence as the main example (Fig. 2), provided that a suitable time-scale is examined. In particular, nestedness and modularity show strongly anti-correlated behavior (*r* = −0.7126), with *z*_*λ*_ peaking when a collective attention gathers around an outstanding topic (the most notable one in this plot being the Superbowl event, between February 3 and 4, 2013).

Finally, to rule out the possibility that our results are specific to socio-political phenomena of the kind of the 15 M movement, we have analyzed an unfiltered dataset of Twitter traffic corresponding to tweets in United Kingdom. As before, bipartite user-hashtag networks are built, but now we chose the subset comprising the top 1,024 most-active users and, independently, the subset of 1,024 most-used hashtags. Note that such independent sampling implies that the corresponding adjacency matrix could be empty –the most active users might not use the most popular hashtags. Results for this dataset show strongly fluctuating patterns for both modularity and nestedness, when measured at large window widths (*w* > 3 h) –not resembling the more persistent, smoothly developed 15 M movement. This is not surprising, as most online topics that succeed in getting collective attention do not demand for days to brew and emerge, but they arise and decay at very fast time scales^4^,^40^. Figure 5 thus shows the results obtained for the UK dataset over a much shorter time scale (*w* = 1 h), revealing that collective attention around certain topics is reached when the network is maximally nested and minimally modular (with overall *r* = –0.7126). Here we do not observe coupled modularity-nestedness regimes (*r* > 0), as the incipient stages of a forming topic go unnoticed in the unfiltered stream. For example, a post hoc inspection of the unfiltered stream revealed the consolidation (but not the incipient stages) of the XLVIII Super Bowl topic, that started on February 3rd, 2013 at 12:30AM CET, showing the highest peak (lowest valley) in the nestedness (modularity) values in the studied period.

## Discussion

In summary, our analyses have unveiled the mechanisms underlying the evolution of an information ecosystem, revealing that there is a traceable pattern for an emerging collective attention event to culminate. Such pattern implies a sudden transition from an initially disperse scenario (modular architecture) to a cohesive situation (nested architecture). Extensive numerical simulations reveal that user-meme mutualistic interactions^9^ drive the networked structure towards that nested-only stage, i.e. the architecture that best accommodates the coexistence of individual participants^12^,^36^. These results stem from an integrated view of the temporal dynamics of emergent collective attention in the context of interdependence and coevolution of human-meme ecosystems, both in online and offline communication.

The present work thus places the study of user-meme structures within the framework of mutualistic communities. This implies that the lessons from such rich tradition can be applied in this new informational context, with the advantage of the finest temporal resolution –time-resolved datasets are scarce in the ecological literature. For instance, the concepts of competition, cooperation and facilitation, vaguely used in reference to information environments, can now be put on firm theoretical standpoints. By connecting meme-mediated human interaction to one of the landmarks in systems ecology –nestedness–, we enlarge the list of complex systems for which such configuration has been reported –with the implications it bears. Such is the case of organizational networks^14^,^16^ or cultural assemblages^32^. Our findings support the idea that nestedness is indeed a dominant pattern in complex networked systems –but it has, paradoxically, received much less attention than modularity. Last but not least, our results provide empirical evidence –at least in the human communication scene– that modularity and nestedness, two dominant architectural principles in complexity, can coexist in a single topology at its early stages, but abruptly bifurcate as the system reaches maturity. Such findings have deep implications on a system, affecting its dynamical properties in terms of diversity, stability, diffusion, and so on. This is then a valuable addition to an ongoing debate about modular and/or nested topologies coexistence, which has mainly occurred in the eco- and biological arena^41^,^42^,^43^ but also, implicitly, on the theoretical one^18^. Our results suggest deep constraints not yet fully understood about network formation and evolution, which perhaps analytical efforts can disentangle in the future. This opens the path to further studies along the lines explored here. Finally, the phenomenology of the transition described in this work suggests that the methodological approach presented here could be applied to other datasets, provided that there is a brewing period in which consensus is built up as time progresses. As such, it cannot describe situations in which unexpected^4^ or exogenous^40^ events (lacking precursory activity) suddenly emerge.

## Materials and Methods

### Data

The analyzed data comes from two disjoint sets of Twitter collections. The data for the Spanish 15 M movement were harvested by a startup company (*Cierzo Ltd*.) for a period of 30 days, starting on April 25, 2011. In that period, protests emerged in Spain in the aftermath of the so-called Arab Spring, with a large demonstration on May 15th and strong echoes up to May 22nd (local elections in Spain). Thus our analysis covers a brewing period with low activity rates (up to May 15th) plus an “explosive” phase beyond that day, decaying beyond May 22nd. Our collection comprises 521,707 messages. The UK collection is not filtered topic-wise in any sense. It comprehends almost 29 million messages for a three month period in 2013, the only restriction being the localization of these tweets: they correspond to messages emitted either from the United Kingdom or Ireland. See section A of the *SM* for details on the events and data collection in both cases.

### Bipartite modular structure

Community analysis is performed *via* Barber’s modularity *Q* maximization. In his work^26^, Barber provides an appropriate null model given the bipartite nature of our networks. In particular, a bipartite network is represented as a block off-diagonal binary matrix:


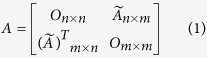


and the adequate null model for it is:


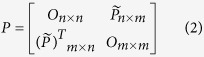


where *O*_*i × j*_ is the all-zero matrix with *i* rows and *j* columns.

All of this is reflected in the magnitude to optimize:


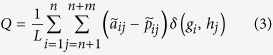


where *L* is the number of interactions (links) in the network, 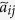 denotes the existence of a link between nodes *i* and *j*, 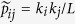 is the probability that a link exists by chance, and *δ* is the Kronecker delta function, which takes the value 1 if nodes *i* and *j* are in the same community, and 0 otherwise. We give some additional details in Section C of the *SM*^44^,^45^,^46^,^47^. Note that the off-diagonal blocks 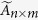 and 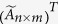 in (1) correspond to **M**_***t***_ and **(M**_***t***_)^T^ respectively. Modularity z-scores (Figs 2 and 5) have been obtained against the average and standard deviation of an ensemble of *Q* for 10^2^ random realizations of **A**^44^.

### Nestedness

In interaction networks, nestedness ref. 48-50, 49, 50, 51, 52 indicates the extent to which specialists interact with proper nested subsets of those species interacting with generalists^12^. Among many methods to quantify nestedness in bipartite networks, here we evaluate it following the spectral approach^27^,^28^,^29^, i.e. the level of nestedness is given by the maximum eigenvalue *λ*_*max*_ of the adjacency matrix **A** of the network. Nestedness z-scores (*z*_*λ*_ in Figs 2 and 5) have been obtained against the average and standard deviation of an ensemble of *λ*_*max*_ for 10^4^ random realizations of **A**. Section B of the *SM* discusses the robustness of the reported results compared to NODF^30^, as well as across the most used of significance tests.

### Mutualistic dynamical model

We model a topic’s evolution integrating the set of differential equations in Bastolla *et al*.^36^ for both classes of nodes on top of synthetic networks, which have been purposefully built to be almost perfectly modular, and almost perfectly nested (see section E.1 of the *SM* for further details). In particular, we consider a mutualistic community consisting of *n* users and *m* different hashtags (memes); the diversity is denoted by *N(t)* and refers to the sum of active users and hashtags at a given moment *t*. Let *U* be the set of users and *H* be the set of hashtags, *s*_*u*_ refers to the relative activity of user *u* and *s*_*h*_ represents the relative frequency of hashtag *h*. In order to model the evolution of the system, we consider that elements of the same group (users or hashtags) are in competition between each other, while they hold a mutualistic relationship with elements of the other group. Therefore, the activity of a given user *u* evolves according to^36^:


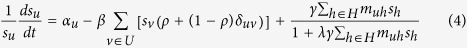


where the first term *α*_*u*_ represents the specific growing rate. The second term of eq. (4) refers to the competition, where *δ*_*uv*_ is the Kronecker’s delta (taking the value 1 when *u = v* and the value 0 otherwise) and the parameter *ρ* modulates the strength of the competition between different users (in correspondence with the biological interspecific competition term). Finally, the third term of eq. (4):


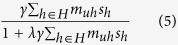


models the mutualism. The user-hashtag interactions are represented through the bipartite graph **M**_*t*_ = {*m*_*uh*_}, where *m*_*uh*_ = 1 if user *u* has posted a message containing the hashtag *h*, and 0 otherwise. *λ* corresponds to the Holling term that imposes a limit to the mutualistic effect, decreasing the mutualistic term to *1*/*λ* for large frequencies. The formula for the evolution of hashtags can be obtained by interchanging the indices of the equation:


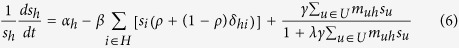


Our aim is to study the evolution of the system in different topologies (nested versus modular) focusing on the survival of memes and hashtags, that is, the diversity in the stationary state. To this end, we performed extensive numerical simulations by integrating the *N* coupled differential equations (4,6) by means of a fourth-order Runge-Kutta method. Section E.2 in the *SM* reports the explored parameter space ref 53 and other details^44^,^45^,^46^,^47^,^48^,^49^,^50^,^51^,^52^,^53^,^54^.

## Additional Information

**How to cite this article**: Borge-Holthoefer, J. *et al*. Emergence of consensus as a modular-to-nested transition in communication dynamics. *Sci. Rep.* **7**, 41673; doi: 10.1038/srep41673 (2017).

**Publisher's note:** Springer Nature remains neutral with regard to jurisdictional claims in published maps and institutional affiliations.

## Supplementary Material

Supplementary Information
