# Supplementary material for: Emergence of consensus as a modular-to-nested transition in communication dynamics
Source: Sci Rep. 2017 Jan 30;7:41673. doi: 10.1038/srep41673 (PMC5278396; doi:10.1038/srep41673)
Supplement: Supplementary Information [file srep41673-s1.pdf]

Supplementary Materials to

Emergence of consensus as a modular-to-nested transition in communication dynamics

Javier Borge-Holthoefer[1,2,3]*, Raquel A. Baños[3], Carlos Gracia-Lázaro[3], Yamir Moreno[3,4,5]*

[1] Qatar Computing Research Institute, HBKU, Doha, Qatar
[2] Internet Interdisciplinary Institute (IN3), Universitat Oberta de Catalunya (UOC), Barcelona, Spain
[3] Institute for Biocomputation and Physics of Complex Systems (BIFI), Universidad de Zaragoza, 50018 Zaragoza, Spain
[4] Department of Theoretical Physics, Faculty of Sciences, Universidad de Zaragoza, Zaragoza 50009, Spain
[5] Institute for Scientific Interchange (ISI), Torino, Italy

* To whom correspondence should be addressed: borge.holthoefer@gmail.com, yamir.moreno@gmail.com

A. Data

The *information ecosystem* under consideration stems from two disjoint sets, which correspond to hashtags and users from the online platform www.twitter.com. Following the analogy with interactions in ecological systems, two types of *species* are considered: users and hashtags (memes). This implies that we do not consider follower/following links between users, nor connections between hashtags due, for example, to co-occurrence in the same tweet. We do not consider either message (tweet) contents, except to extract the hashtags in it: our final aim is to keep record of who-said-what in terms of agents using hashtags.

Spanish collection. The collection from Spain comprises 521,707 tweets containing at least one hashtag, 22,375 unique hashtags and 78,080 unique users. The observation period ranges from the 25th of April at 00:03:26 to the 26th of May at 23:59:55, 2011, and data was collected by selecting all the tweets containing at least one of a preselected set of 70 hashtags related to the 15M movement with the aim of filtering out only the activity related with this topic (see Table S3). Data collection was carried out by the start-up Spanish company *Cierzo Development Ltd*.

UK collection. The UK collection comprises 28,928,528 tweets emitted by a set of 842,745 unique users between the 18th of January at 18:41:56, to the 31st of May at 22:41:56, 2013. The set of unique hashtags in this case is 2,196,934. Unlike the case of the Spanish dataset, tweets have been filtered by selecting only those that are geolocalized in the United Kingdom and Ireland. In this way, this set provides a raw dataset (only limited by geolocalization) of twitter traffic in which the activity is not filtered by topic, hashtags or users. See Table S4 for a glimpse on the most common hashtags.

A.1. Data as an evolving bipartite graph

As it is clear in the main text, we attempt to account how the user-hashtag ecosystem changes over time. To this end, we build a sequence of snapshots out of the data. These snapshots have an arbitrary window

width w, and adjacent snapshots have an overlap of φw. Such overlapping scheme is a rather standard procedure when considering chunked temporally-resolved information, to provide a smooth account of change in time.

The question remains how these datasets can be suitably represented. The most natural way to map user-hashtag interactions is through a bipartite graph of relations, which in turn corresponds to a rectangular presence-absence matrix $\mathbf{M}_t = \{m_{uh}\}$, where $m_{uh} = 1$ if user u has posted a message containing h, and 0 otherwise (note that matrix $\mathbf{M}_t$ corresponds to block $\tilde{\mathbf{A}}_{n \times m}$ of the block off-diagonal binary matrix $\mathbf{A}$ in eq. (1) of the main text). Noteworthy, this implies that only binary values are considered, i.e. the number of interactions between nodes u, h is not recorded. Besides, we acknowledge that results in the main text are not affected by the chosen window width (results there correspond to w = 12 hours and 3 days, with overlaps of 6 and 36 hours, respectively). See below for more details.

It is also important to highlight that the $\mathbf{M}_t$ matrices may not contain the same nodes across t: as time advances, users join (disappear) as they start (cease) to show activity; the same applies for hashtags, which might or might not be in the focus of attention of users. This volatile situation is quite normal in time-resolved ecology field studies [33, 35, 53], where the accent is placed on the system's dynamics – rather than individual species. Moreover, the level of turnover in the sequence of data is very informative, as it characterizes how the system renews its structure over time (see the main text).

A.2 Pruning the data

The large size of our two datasets –78,081 unique users and 22,376 unique hashtags in the 15M dataset, and 842,745 users plus 4,217,530 hashtags in the UK dataset– handicaps the data processing and makes the calculations time-consuming. We must therefore apply some restrictions to the number of users and hashtags considered in the network.

Therefore we apply a rather straightforward criterion, by which we prune the least active users in the data. This means that only top-contributors (and their associated hashtags) show up in the matrices that we study. In doing so, we guarantee that the whole approach makes sense: only by including the most active users we make sure that generalists and specialists will show up –if any nested patterns are to be found. Also the probability of obtaining a connected matrix is higher. Again, we acknowledge that ours is an arbitrary decision. To provide solid evidence, we have tried several matrix sizes.

Spanish dataset. Whereas results reported in the main text are based on the 1,024 most active users, we have also tested smaller sets with qualitatively the same results (see Figure S1). In this Figure, we represent the standardized results for both nestedness (left) and modularity (right). Both magnitudes will be described in details later (section B and C). Three dates are also considered at different moments of the 15M movement: three days before the main camps took place –May 12th–, at the onset of the protests; May 15th itself; and May 19th, when the maximum nestedness is achieved and protests are considered to have reached high levels of visibility. Nestedness curves show a tendency to saturate for

large values of the number of users selected. This flattening is achieved at lower values for earlier dates, being far from saturation on May 19th. In view of these results, we can safely conclude that our pruning procedure, i.e., the restriction to the most active users, does not give rise to a misleading claim about the nested structure organized around the movement formation. So far, and to avoid extrapolation, we can safely state from Fig. S1 that, if we build a nestedness time series and a modularity time series (admittedly both quite poor: only 3 points each) we see that 19M shows, nestedness-wise, a maximum, regardless of the size one wishes to pick; and modularity-wise a minimum, regardless of the size on wishes to pick (except for very small sizes, $n < 150$). In summary, for any size reported in Fig. S1,

$$z_N^{12M} < z_N^{15M} < z_N^{19M}$$

and

$$Q^{12M} > Q^{15M} > Q^{19M}$$

which would render perfect anti-correlation (that is, a stronger result than the one reported in the main text).

UK dataset. For this dataset, the filter is applied in a slightly different way: the cutoff is applied to both users and hashtags, by choosing the 512 more active users and the 512 most-used hashtags. The reason underlying the additional constraint on hashtags and the smaller number of nodes considered, is the large amount of hashtags used in this dataset: 1,024 users can generate from 2,245 to 13,113 hashtags, depending on the observation time window. Some technical details about the observation period, number of users and hashtags, and time-windows width can be found in Table S1.

As for how we build bipartite networks for the UK dataset, different possibilities arise: on the one hand, we could randomly select a subset of users and hashtags involved in the network, but in this way we might be missing the relevant agents thought to play a major role in the contribution to the nestedness of the whole system. Besides, a random selection could lead to empty matrices (none of the selected users tweeted any of the selected hashtags). We must, nevertheless, remark that this situation is highly unlikely for the 15M event, as a result of the very nature of the dataset: only people and hashtags related to this particular topic were extracted from Twitter. We will be making use of this method as a way to compare the nestedness levels in the 15M with a topic null model, built from data from the UK dataset.

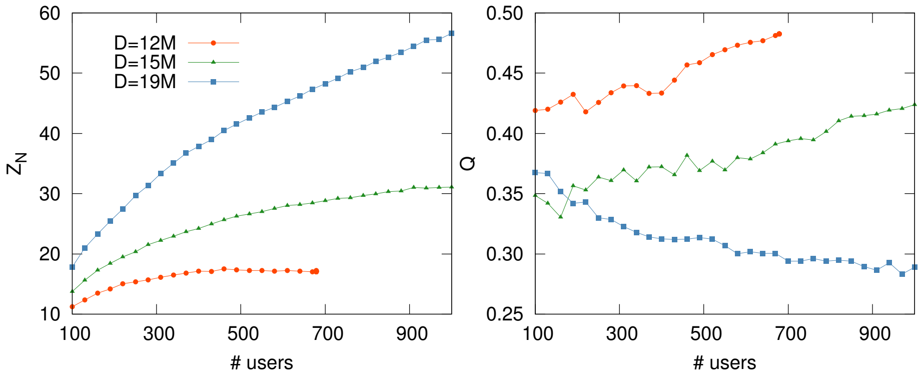

Figure S1: Robustness against matrix size. For the w = 12h set some days have been selected. We perform the null model analysis for different cutoffs in the number of users (x axis), and show how the standardised leading eigenvalue (left) and the standardised modularity (right) evolve. The end at ~700 users for the curve corresponding to D = 12M indicates that the largest possible matrix has been reached, i.e there are no more active users at that particular day. These results not only guarantee that our conclusions about the nested structure around the 15M are robust, but also show that the observed peak would be more prominent if we considered the real matrix including all the users and hashtags.

	15M, 2011	UK, 2013	UK (inset), 2013
Date range	25/04 to 26/05	18/01 to 31/05	31/01 to 06/02
# total users	78,081	842,745	122,553
# total hashtags	22,376	4,217,530	264,291
Filter to users	1,024	1,024	512
Filter to hashtags	--	1,024	512
Final # users	17,202	50,091	37,174
Final # hashtags	12,384	19,905	22,933
Time windows	12h; 72h	24h	60min; 120min
Overlap	6h; 36h	12h	30min; 60min

Table S1: Datasets summary details. The date range, number of total users and hashtags are displayed. We also indicate the cutoff in the number of users and hashtags (if any) that has been applied. An unspecified hashtag filter indicates that the hashtag set is determined by the set of selected users. Users are filtered by activity and hashtags by usage. We also show the final number of users and hashtags after the selection process. Finally, the window width and overlap between consecutive windows are also displayed.

B. Nestedness in online social networks

Robustness across metrics. Several studies have been focused on quantifying nestedness, the first proposals being made by Hultén [49], Darlington [46] and Daubenmire [47] to describe patterns in which species-poor sites are proper subsets of those ones present at species-rich sites. Nestedness analysis has become very popular among ecologists, and, although the concept is widely accepted, it has not been formally defined, yielding to several distinct metrics [29,30,45]. In this work (main text), we adopt a definition numerically confirmed by Staniczenko et al. [29], where nestedness is given by the maximum eigenvalue of the network's adjacency matrix. This metric is based on a theorem regarding chain graphs first provided by Bell et al. [27,28], where it is shown that among all the connected bipartite graphs with N nodes and E edges, a perfectly nested graph gives the larger spectral radius. The

method is advantageous over other possibilities due to the invariance of eigenvalues under matrix permutations, and the remarkably low computation time required to perform eigenvalue calculations, even for large matrices. This is an important detail provided that z-scores for nestedness are obtained against 10^4 random realizations.

Nevertheless, we have checked the validity of our results against the improved metric NODF, defined by Almeida-Neto *et al*. [30]. This measure is based on two simple properties: decreasing fill (DF) and paired overlap (PO). Assuming that row (column) i is located at an upper position in the sorted presence-absence matrix from row (column) j, the decreasing fill condition imposes that a pair of rows (columns) can only contribute to the nestedness if the marginal total –the number of interactions a row (column) has– of row (column) i, is greater or equal to the marginal total of row (column) j. In this case, the paired nestedness, N_{ij}, is equal to the paired overlap PO_{ij}, i.e., the number of shared interactions between rows (columns) i, j. The metric can be summarized as:

$$\mathrm{NODF} = \frac{\sum_{ij} N_{ij}}{\frac{m(m-1)}{2} + \frac{n(n-1)}{2}} \qquad (1)$$

where

$$\begin{aligned} N_{ij} &= 0 \quad && \text{if} \quad \mathrm{MT}_i < \mathrm{MT}_j \\ N_{ij} &= \mathrm{PO}_{ij} \quad && \text{if} \quad \mathrm{MT}_i \geq \mathrm{MT}_j \end{aligned} \qquad (2)$$

Both metrics are compared in Figure S2. In the x-axis the standardized value of the leading eigenvector is displayed against the standardized NODF measure. Matrices involved in the plot correspond to graphs at the distinct snapshots with time-window w = 1d. These results are displayed along with the Pearson and Spearman coefficients and their p-values, showing a good linear correlation with p-values $p < 10^{-3}$.

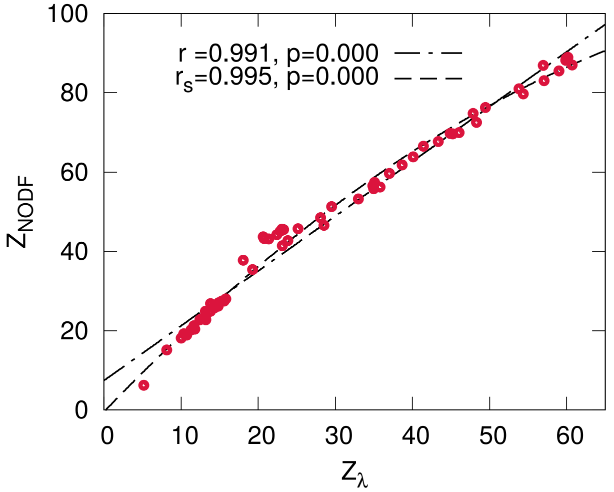

Figure S2: Comparison against nestedness metrics. For every matrix from the set of time windows with w = 12h, the standardised leading eigenvalue, z_λ, and the standardised NODF metric, z_{NODF}, are computed. There is a good agreement between both metrics, as the Pearson coefficient, r, and the Spearman coefficient, r_s, show along with their p-values.

Robustness across significance tests. The fact that real matrices are usually far from being perfectly nested, imposes the use of a test for the significance of nestedness values. Such a test implies the implementation of a null model and the computation of standardized results, and additionally, allows one to compare matrices with distinct sizes, this comparison being impossible otherwise. Regarding

modularity, the metric already includes in its very definition a null model, in such a way that the modularity obtained is already a comparison with a randomized counterpart of the network.

Different null models may be proposed. For example, one could think of a null model rewiring the set of links present in the network. A strict application of such scheme would not maintain the bipartite structure of the network, and for that reason it should be avoided.

Within this restriction we can still think of some variations. Here we explore two different possibilities, as discussed in [41]. In null model I, the number of links in the network is preserved, but placed at random within the matrix –although respecting the class of the origin and end of it. The degree sequence is therefore not preserved. Null model II is a probabilistic null model where an interaction between hashtag *h* and user *u* is established with probability proportional to their connectivity,

$$p_{uh} = p_{hu} = \frac{1}{2}\left(\frac{k_h}{m} + \frac{k_u}{n}\right) \quad (3)$$

In the above expression, *n* stands for the total number of users, i.e., the first dimension of M_{uh}, and m for the number of hashtag, equal to the second dimension of M_{uh}. k_u and k_h correspond to the degree of user *u* and hashtag *h*, respectively. This model maintains the number of interactions per class only approximately, i.e. it probabilistically maintains the observed total number of interactions.

We can go further and consider an X-swap scheme null model III, in which a rewiring of the edges is applied but keeping constant the degree sequence of the nodes in the system. This null model, however, is too restrictive, and gives a small number of possible configurations, specially for those matrices having few non-empty cells. We must consider null models having a balance between the number of possible configurations and strictness. For this reason we choose to discard null model III, and apply the probabilistic null model II, which is the strictest between models I and II. Figure S3 reports the consistency of the results for the nestedness using either of the chosen null models. Z-scores have been calculated over 10,000 randomizations.

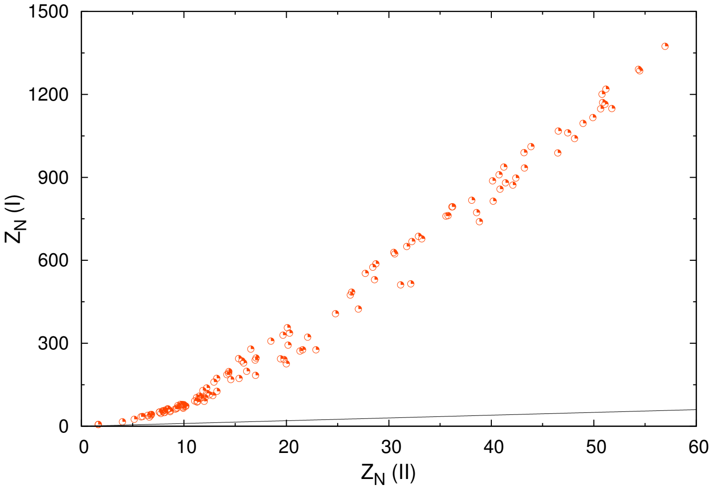

Figure S3: Robustness against statistical null models for nestedness. Unsurprisingly, results for the null model I (much less restrictive) yield extremely high z-scores, as opposed to the comparatively moderate results from null model II (note the y=x line as a visual aid). Despite these differences, both models are highly consistent at quantifying the level of significance for the nestedness.

C. Modularity

Modularity was originally proposed as a metric for community detection in networks by Newman and Girvan [51] aiming at identifying the mesoscale organization of networks, which reveals many hidden features invisible from a global perspective of the network; informally, modularity (typically labeled Q) relies on the detection of densely connected subgraphs: it quantifies the extent to which nodes in a network tend to cluster together, in comparison to the expected distribution of a random counterpart (null model).

One of the interesting aspects of Q is its reliance on the concept of null model, which can be taken as the baseline against which optimization makes sense. This has allowed the original formulation by Newman to be extended to other scenarios, namely directed, weighted or signed (if we pay attention to the features of the links); and bipartite and multiplex networks, beyond the (more common) unipartite networks. The general layout of Q is

$$Q = \frac{1}{2m} \sum_{i}^{N} \sum_{j}^{N} \left(A_{ij} - P_{ij}\right) \delta\left(g_i, g_j\right) , \qquad (4)$$

where g_i represents the module node i belongs to, A_{ij} is the real adjacency matrix of the network, and P_{ij} are the probabilities that an edge linking nodes i and j exists in the null model.

The key point is to define, in this equation, a suitable, adapted null model to confront the real connectivity patterns (as in the mentioned cases). The issue is controversial because even within a type of network different possible null models can be defined. In the bipartite scheme we find two main proposals. We have chosen to work with Barber's definition of modularity for bipartite networks [26] (see also the main text), ruling out the proposal by Guimerà *et al*. [48]. In Guimerà's proposal, modules are forced to be defined strictly in class "purity", that is, a module can only contain nodes of a single class. His method is thus almost equivalent to optimize modularity on the projected unipartite network, which collapses the information in the bipartite original network onto one of its classes (see [48] for the details). On the contrary, Barber's definition naturally incorporates combined (or mixed) modules, formed by nodes from both classes.

The choice of one or another definition is a matter of the problem one intends to solve. Indeed, it may not make a lot of sense to define movie-actor mixed modules, because the semantics of such a module is not very clear. In other problems, however, it may be more convenient to allow for mixed modules. This is often the case in ecology (as for instance in [41]), because it is more interesting to identify modules that have a precise biological meaning as potential co-evolutionary groups [54] or as cores of mutualistic networks [9]. As we are also, in our user-meme systems, more interested in this co-evolutionary perspective, we have taken Barber's approach.

We have applied this metric making use of the software provided by Marquitti *et al*. [50], where the simulated annealing method [27] is used to maximize Barber's modularity. Statistical significance of the results is checked obtaining the z-score of the original network modularity, against the average and standard deviation of 100 random realizations (null model II as for nestedness, see above).

D. Nestedness and Modularity: further considerations

Robustness across window widths. Beyond assessing the robustness of the results for the nestedness values (regarding the used metrics and null models), we also need to test for robustness against the (admittedly arbitrary) choice of a window-width. This applies both to the soundness of the results in nestedness and modularity.

In Figure S4 we report results for modularity and nestedness (both in their standardised version) for every width w we have tested. Upon inspection, it is clear that results are noisier the narrower the window is –the regularity of the peaks suggests that the measures are sensitive to circadian rhythms (periodic temporal patterns). For values aggregating the activity for one day and beyond, such periodic variations disappear. Remarkably, nestedness (lower panel) shows the same trend for every window width. In contrast, the trajectory of the modularity z-scores is coherent up to w = 1 day, but it is blurred out for w = 3 days. These results (together with those obtained for the UK dataset) suggest that events have their very own characteristic timescale [55], and observed trends are valid only within a relatively precise range.

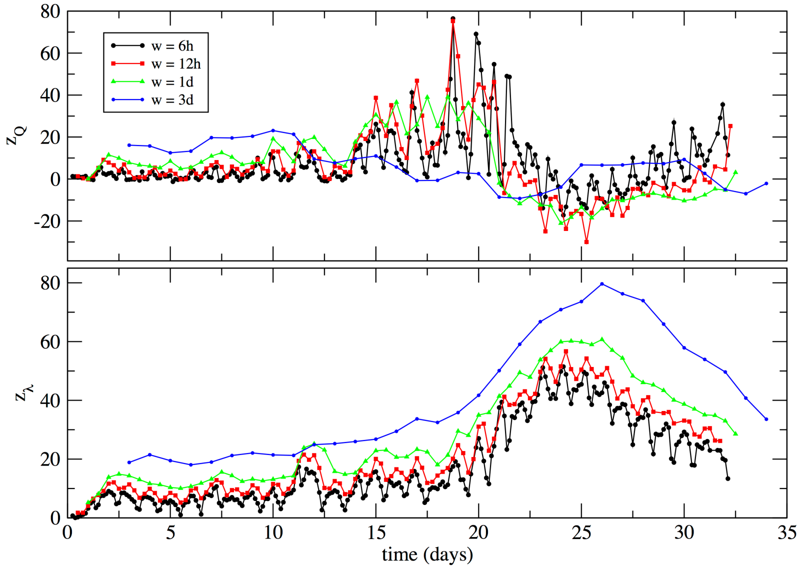

Figure S4: Robustness against window width. Standardised nestedness, z_λ , and modularity, z_Q, values are displayed for window sizes 6h, 12h, 24h and 72h.

We observe the same robustness in the UK datasets for a window width of w = 2 hours (as opposed to w = 1 hour reported in the main text). Given the fast time scale of the event (it fully develops in less than two days), wider window schemes blur the results.

Ruling out epiphenomenal conclusions. Both in the main text and throughout this document we have provided solid evidence that, in an information ecosystem such as Twitter, topics arise in a nested scheme out of an initially modular structure. One may argue, however, that this striking outcome may be artificial in different senses. First, it is possible that the modular-to-nested transition occurs out of a "topological artifact", namely, that the network starts as a broken set of small components (thus being trivially modular) and undergoes a percolation process such that nestedness is possible from then on. In Figure S5 the size of the giant component of the system is plotted as it evolves in time. Such component is always above 0.78N, and as such a percolation transition is never observed.

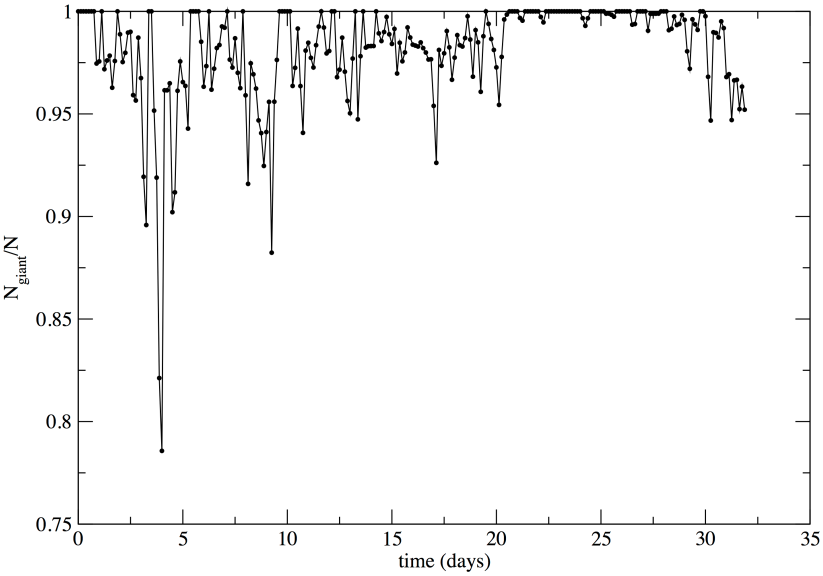

Figure S5: Evolution of the size of the giant connected component (as a proportion of the total size of the system). Notably, the *y*-axis is labeled from 0.75 and above, which implies that, for the whole time range (over a month), the system does not undergo any abrupt percolation process. The figure corresponds to a window width w = 6 hours (the noisiest and sparsest one).

A second consideration implies our disregarding of weighted values. Indeed, we have focused on binary, presence/absence matrices –in an effort to follow the ecosystems literature. Additionally, NODF does not have, to our knowledge, a weighted equivalent, so comparison is properly done only with a binary representation of the system.

Admittedly, this represents a loss of information, which could potentially affect the results. We are aware that the spectral radius approach to nestedness does allow for weights to be present in the interaction matrix. For the sake of completeness, we have measured nestedness also considering weights, which stand for the frequency with which an individual used a certain hashtag, given a certain time window. The result can be seen in Figure S6, where the growing pattern follows precisely the trends reported in the main text (Figure 2) and here (Figure S4).

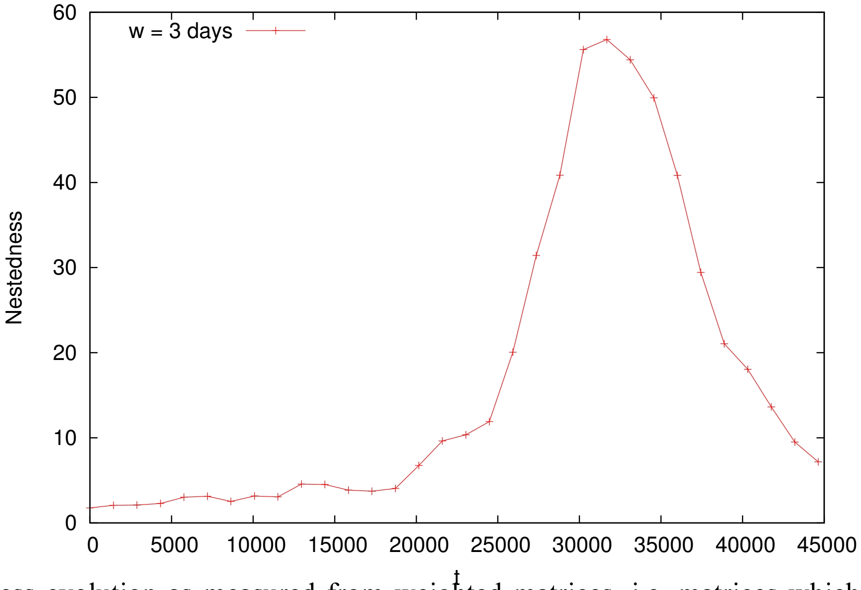

Figure S6: Nestedness evolution as measured from weighted matrices, i.e. matrices which encode the absolute usage counts of hashtags by the corresponding Twitter users. The figure corresponds to a window width w = 3, and delivers the same growing patterns as its own binary counterpart. (time in the x-axis is expressed here in minutes since the origin of our data; here, t = 30000 is roughly equivalent to May15th).

E. Mutualistic Dynamical Model

According to the dynamical framework from Bastolla *et al.* [36], the evolution of a mutualistic ecosystem can be modeled through a set of N differential equations in which each equation represents the variation of the frequency of a plant or a pollinator. Competitive interactions are estimated through linear function responses, $-\beta_{ij}^{(P)}N_j^{(P)}$ for plants and $-\beta_{kl}^{(A)} N_l^{(A)}$ for animals, where competition matrices $\beta_{ij}^{(P,A)}$ are symmetric and non-negative. In the same way, mutualistic interactions between plants and pollinators are modeled through non-linear functional responses of Holling Type, $f(N) = \gamma N/(1+h\gamma N)$, where mutualistic relationships are described through two symmetric and non-negative matrices $\gamma_{ij}^{(P,A)}$ and the Holling term h imposes a limit to the mutualistic growth rate, avoiding divergences in the case of large populations. The equations for the system's dynamics are described in *Materials and Methods* section of the main text.

E.1. Synthetic topologies

Regarding topologies, we built *ad hoc* two ensembles of synthetic networks for different network sizes. For each pair of ensembles (equal size, equal link density), the networks of the first ensemble present an almost perfectly nested architecture, while the networks of the second one exhibit an almost perfectly modular structure. All the networks of a given size N were built with the same number of users and hashtags $n = m$. Nested networks were constructed starting from a perfect nested structure, involving a connectivity distribution $k_u = u$, $u = 1, 2, \ldots$; $k_h = h$, $h = 1,2,\ldots$, and subsequently randomizing each link with probability $p = 0.02$. This method provides networks with an almost perfect nested topology and

mean connectivity <k> = $N/4$.

According to the procedure by Newman [51], modular networks were constructed starting from a perfect modular structure consisting of 5 disconnected blocks (cliques) of equal size $N_i = N/5$, and subsequently randomly connecting pairs to reach the connectivity <k>= $N/4$. The number of blocks (5) and the rewiring probability $p = 0.02$ were arbitrarily chosen. Nevertheless, the results are robust against variations of these values, as shown in figure S7.

E.2. Realizations

In each realization, we used a different network of the corresponding ensemble, and assigned different random initial frequencies to each user and hashtag in the interval $s_{u,h}(t = 0) \in (0, 1)$, and different growing rates in the interval $\alpha_{u,h} \in (0.85, 1.1)$. We ran the dynamics defined by equations (4) and (6) of the main text and, once the stationary state was reached, we computed the survival rate by adding the number of users and hashtags with frequency $s_{u,h} > 0$ and then divided by their initial number N. Accordingly, the survival area stands for the region of the parameters space with a survival rate greater than a given value. We performed 1000 different realizations per each point of the space of parameters β × γ and for each size and topology. According to the standard biology procedures (see, e.g., [36, 54]), the inter-specific term was fixed to $\rho = 0.2$ and the Holling term was set to $\lambda = 0.1$. The values for the competition and mutualistic terms covered the range $\beta,\gamma \in [0.1]$ with intervals of δ_β , $\delta_\gamma = 0.05$ (from weak to strong mutualism regimes).

Results of these extensive simulations are shown in Figure 3 of the main text, where left panels represent the survival rate (i.e., the diversity in the stationary state) as a function of the competitive and mutualistic terms β and γ, for a system size of $N = 1000$. Right panels of Figure 3 of the main text represent the normalized area of the space of parameters $\beta \times \gamma$ that exhibits a survival rate equal o higher than the corresponding value of the x-axis, for different network sizes in different panels: $N = 50$, 100, 300, 1000. As discussed in the main text, a large area of the space of parameters exhibits high persistence for the nested architecture where persistence is low for the modular one, *but never the opposite*. Otherwise, for the modular architecture, the area of the space $\beta \times \gamma$ with a given persistence decreases sharply with the network size, while for the nested architecture this dependence is smaller. This effect saturates for large values of the network size, that is, once the size $N \sim 500$ is reached, the size of the network does not have a noticeable effect on persistence anymore. Figures S8 and S9 complement, respectively, panel left and right of Figure 3 of the main text. Figure S8 represents the persistence (i.e. diversity of memes and hashtags in the stationary state) for each value of β and γ, for nested and modular networks of 100, 500, and 1000 nodes. Additionally, Figure S9 represents the normalized area of the surface ($\beta \times \gamma$) that exhibits a persistence equal o higher than a given value as a function of that value, for nested and modular networks. In the above results (Figure 3 of the main text and Figures S6-S9), the interval $\alpha_{u,h} \in (0.85, 1.1)$ has been taken according to the biological literature (see, e.g. [36]). Nevertheless, the main result (nested architectures out-survive modular ones) holds for wider interval of $\alpha_{u,h,}$ as shown in figure S11 for $\alpha_{u,h} \in (0.5, 1.5)$.

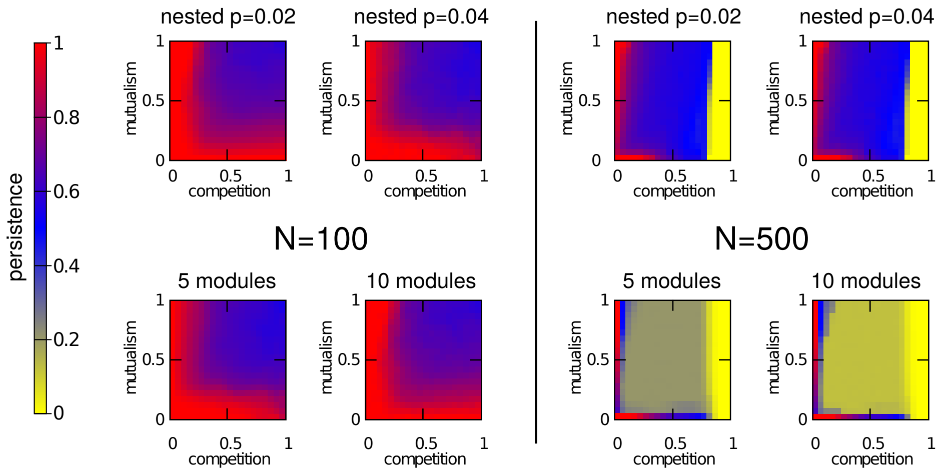

Figure S7: Survival rate as a function of β and γ, for different values of the rewiring probability p in the nested networks (up panels) and different number of modules in the modular networks (down panels). Left panels correspond to a network size of $N = 100$, while right panels correspond to $N = 500$. Each point is averaged over 1000 different initial conditions.

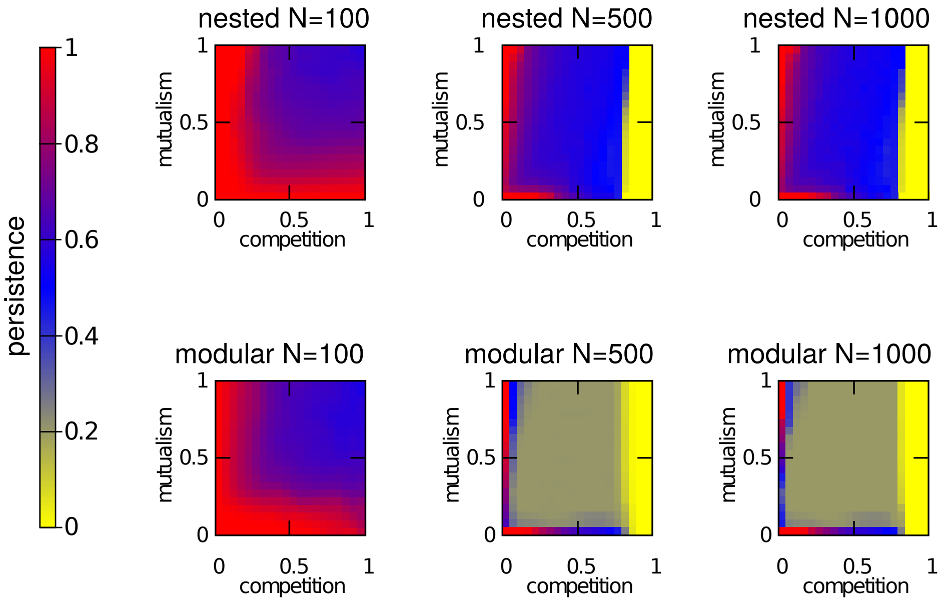

Figure S8: Survival rate as a function of the competition β and mutualism γ terms. Upper (resp., lower) panels correspond to a nested (resp., modular) architecture. Each column corresponds to a different value of the network size: $N = 100$ (left), $N = 500$ (center), $N = 1000$ (right). Each point is averaged over 1000 different initial conditions.

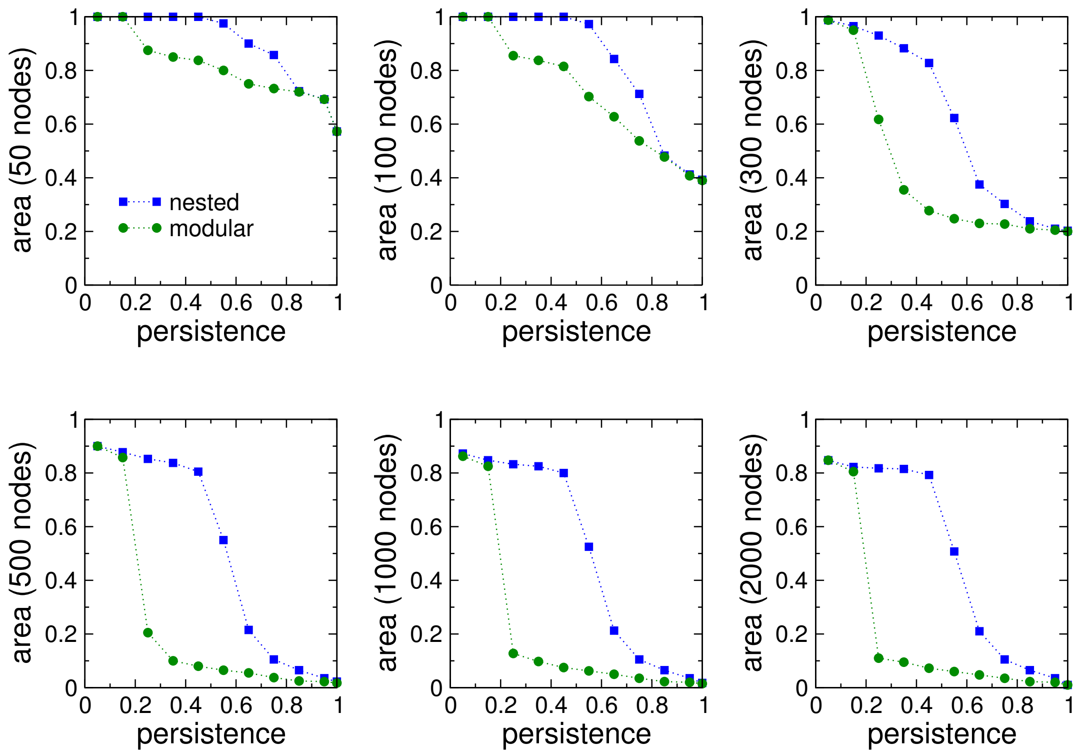

Figure S9: Normalized area of the space $\beta \times \gamma$ with a survival rate equal o higher than the corresponding value of the x-axis. Different panels correspond to different values of the network size: N = 50, 100, 300, 500, 1000, 2000. Note that, for the sake of comparison, 4 of the panels are merely reproducing those reported in Figure 3 of the main text.

The value ρ = 0.2 in the simulations from Figure 3 is taken from the ecological literature, where the intra-species competitive term (1 - ρ) is usually considered to be greater than the inter-species term (ρ), as members of the same species are competing for the same resources. For the sake of completeness, we have studied as well the case N = 1000, ρ = 0.6, which in our study corresponds to an inter-users stress greater than the intra-users stress. Figure S10 shows that, when the inter-users stress exceeds the intra-users stress, the main feature observed in the dynamics remains intact, namely, modular networks exhibit poor survival, while nested networks show equal or higher levels than the modular structure in any given region.

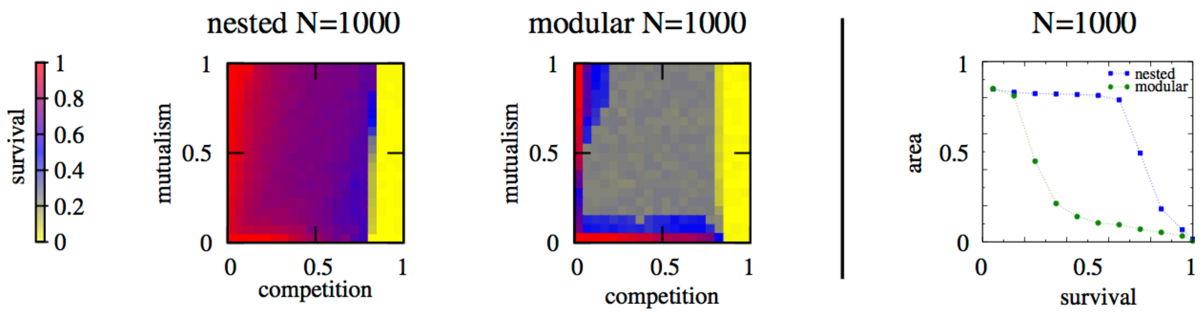

Figure S10: Survival rate (left) and normalized area survival (right) for a network with N=1000 and ρ = 0.6 (corresponding to Figure 3 of the MT, and Figures S8 and S9 of the Supplementary Materials).

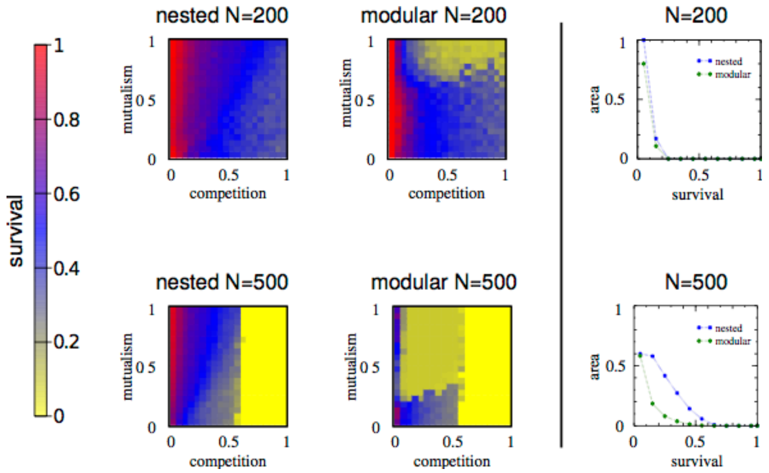

Figure S11: Left and center panels show the survival rate (color code) as a function of competition (x-axis) and mutualism (y-axis). Left (resp., center) panels correspond to a nested (resp., modular) architecture. Different raws correspond to different values of the network size: N = 200, 500. Right panels show the normalized area of the space $\beta \times \gamma$ with a survival rate equal o higher than the corresponding value of the x-axis. $\alpha_{u,h} \in (0.5, 1.5)$.

F. Core-periphery structure

Meso-scale structures in networks have received considerable attention in recent years, as the detection of these intermediate-scale patterns can reveal important characteristics that are hidden at both local and global scales. Among the wide diversity of methods aiming at the detection of such structures, community detection methods have become very popular and successful. In this section we focus our attention on a different type of meso-scale structure, known as the *core-periphery* structure, that helps one to visualize which nodes of the graph belong to a densely connected component or *core*, and which of them are part of the network's sparsely connected *periphery*. Nodes belonging to the core should be relatively well connected to other nodes in the network, either central or peripheral; whereas nodes in the periphery should be those elements poorly connected with the core, and disconnected from the periphery. According to this intuitive notion, many methods have been proposed. We follow here a method developed by Della Rossa *et al.* [38], based on the profile derived by a standard random walk model. It and can be obtained in a very general framework and is applied here for undirected unweighted networks.

Let $w_{ij} = w_{ji}$ be the link of weight 1 between nodes $i \leftrightarrow j$ in our network of size N. At each time step, the probability that the random walker at node i jumps to node j is given by m_{ij}:

$$m_{ij} = \frac{w_{ij}}{\sum_h w_{ih}} = \frac{1}{k_i} \qquad (5)$$

where k_i is the degree of i. The asymptotic probability of visiting node i has the closed form

$$\pi_i = \frac{k_i}{\sum_j k_j} \tag{6}$$

The method starts by randomly selecting a node i among those with the weakest connectivities, and assigning $\alpha_i = 0$. Pk, the set of nodes that are already assigned at step k, is then filled with i, $P_1 = \{i\}$. For the following steps, $k = 2, 3, ..., n$, the node j attaining the minimum in

$$\begin{aligned}\alpha_j &= \min_{h \in N \setminus P_{k-1}} \frac{\sum_{i,j \in P_{k-1} \cup \{h\}} \pi_i m_{ij}}{\sum_{i \in P_{k-1} \cup \{h\}} \pi_i} \\ &= \min_{h \in N \setminus P_{k-1}} \frac{\sum_{i,j \in P_{k-1}} \pi_i m_{ij} + \sum_{i \in P_{k-1}} (\pi_i m_{ih} + \pi_h m_{hi})}{\sum_{i \in P_{k-1}} \pi_i + \pi_h}\end{aligned} \tag{7}$$

is selected. If it is not unique, a randomly chosen node among them, l, is selected, and $P_k = P_{k-1} \cup l$. Although the algorithm presents some randomness, it has been verified that the effect in the analysis of real-world networks is negligible. The core-periphery profile is then the set $\{\alpha_k\}$, with $0 \le \alpha_k \le 1$,where $\alpha_k = 0$ for nodes belonging to the periphery and $\alpha_k > 0$ for nodes in the core.

As the goal of this section is to identify the possible formation of a core during the days preceding the 15M, a distance metric should be defined. As a first approach we consider the distance between two core-periphery structures as the product between the two $\{\alpha_k\}$ sequences,

$$\vec{\alpha}_1 \cdot \vec{\alpha}_2 = \sum_{i=0}^{n \times m - 1} \alpha_{1i} \alpha_{2i} \tag{8}$$

Notice that the α "vectors" do not necessarily share the same coordinates, that is, it may happen that a given node (now by nodes we refer to users or hashtags indifferently) present in $\vec{\alpha}_1$ does not appear in $\vec{\alpha}_2$ because it was not part of the network. Whenever this is the case, we consider the contribution to the dot product to be zero (i.e., as if it were at the periphery). On the other hand, we normalize the above expression in order to get a bounded value: $0 < \vec{\alpha}_1 \cdot \vec{\alpha}_2 < 1$,

$$\vec{\alpha}_1 \cdot \vec{\alpha}_2 = \frac{1}{\|\alpha_1\| \|\alpha_2\|} \sum_{i=0}^{n \times m - 1} \alpha_{1i} \alpha_{2i} \tag{9}$$

which is the expression used in the main text and labeled as D_{RC}.

In the main text we have discussed the conformation of a relatively stable core of hashtags around the 15M day, in contrast to a high turnover of users coming to and leaving the core at different snapshots. Here, we scrutinize further such a finding by ruling out the possibility that it could be due, for example, to the fact that the set of users in the core could be similar over the distinct time-windows and change abruptly at the reference point under consideration. To this aim, we additionally measured the distance of a given core C_t from the previous core C_{t-1} –the core present in the previous time-stamp. Results in Figure S12 reject this conjecture: the turnover of users is still high –the distance is small– when the core

is compared with that in the preceding graph, suggesting that users are actually entering and exiting the key positions in the network. In contrast, hashtags keep relatively constant at high distances, indicating that the core near the 15M is formed smoothly –the exception to this being a sharp decrease around the 15M if observed at a 3-day window resolution. The reason for this behavior is the takeover of new hashtags (with respect to the ones that originated the protest), which pushes the original ones away from the core of the structure. The best example of this is the hashtag *#democraciarealya* ("real democracy now"), which is placed at the core of the bipartite network for a long period of time, but its leading role is substituted by the more generic (and "cheap" from a microblogging perspective) *#15m* from the immediately previous days of May 15 and onwards.

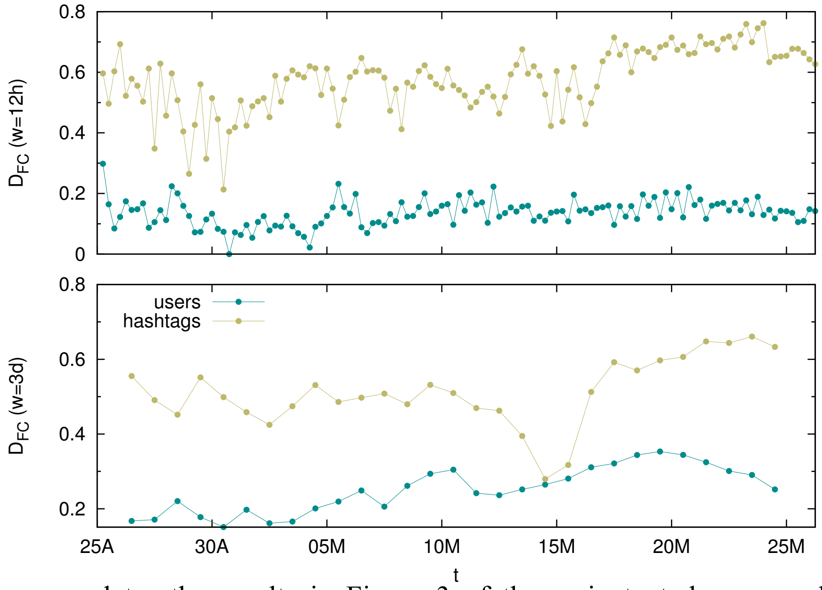

Figure S12: The figure emulates the results in Figure 3 of the main text; however, distances are computed between each time stamped core and the former configuration, C_t vs. C_{t-1}. As in the main text, the figure illustrates that hashtags build a more stable core in comparison to users.

G. Anti-correlation between nestedness and modularity

Further details on the reported Q-nestedness correlated/anti-correlated patterns can be seen in Table S2 and Figure S13.

Dataset		*r*		*p*-value
15M	w = 6h	pre May 15	0.8182	10^{-5}
		post May 15	-0.7569	10^{-5}
	w = 12 h	pre May 15	0.8179	10^{-5}
		post May 15	-0.8023	10^{-5}
	w = 24 h	pre May 15	0.7997	10^{-4}
		post May 15	-0.7819	10^{-5}
	w = 72 h	pre May 15	0.5438	not significant
		post May 15	-0.2732	not significant
UK	w = 1h	–0.7126		10^{-5}

Table S2: Pearson correlation coefficient values for nestedness and modularity, along with their *p*-values, for both datasets and available window widths. In the case of 15M, we report Pearson correlations before and after the climax of the event (though the exact moment at which modularity abruptly collapses is slightly different in each case, see panels in Figure S13). Note that correlations fail to be significant (that is, $p > 0.05$) for $w = 3$ days, as the results for modularity are blurred compared to the observed pattern in $6\text{h} \leq w \leq 24\text{h}$.

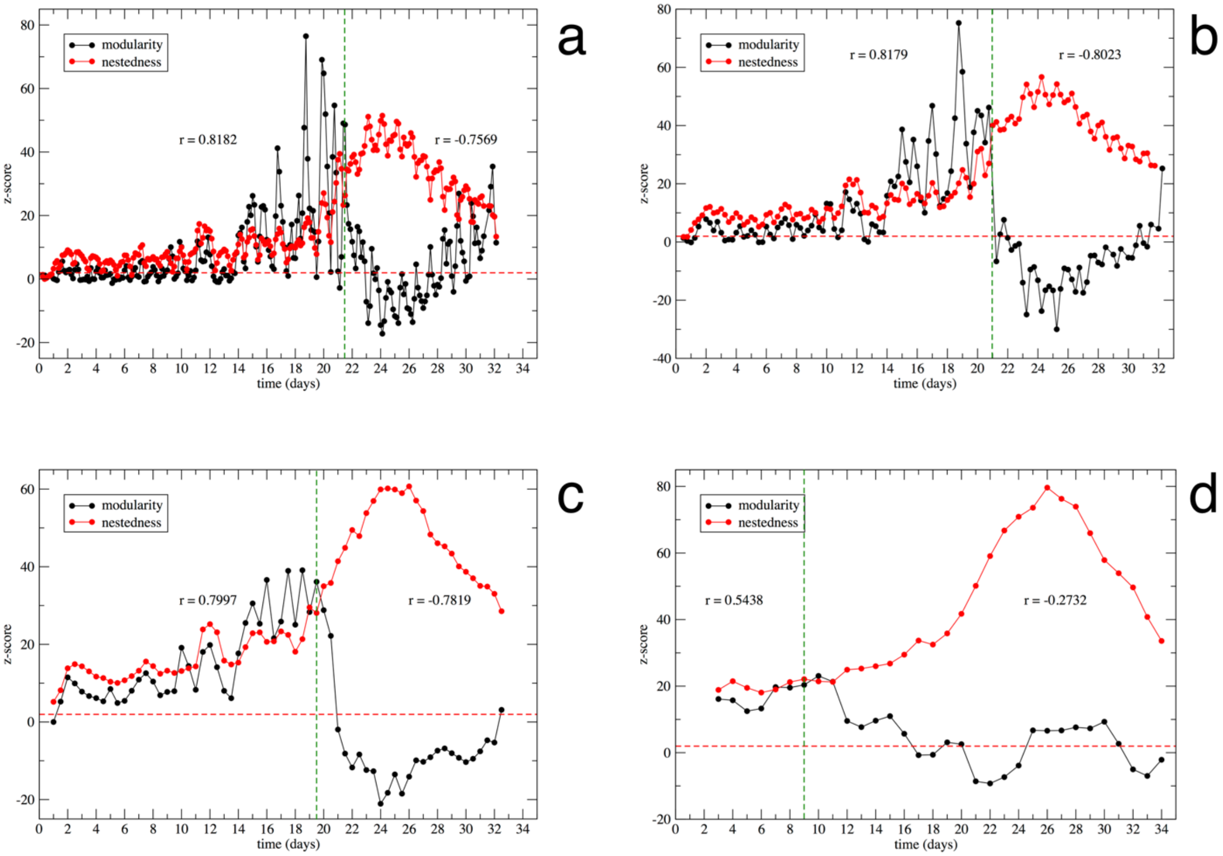

Figure S13. Evolution in time of nestedness and modularity z-scores for all the window widths (6h, 12h, 24h and 72h) in the 15M dataset (c panel is the same on as reported in the main text, and repeated here for the sake of comparison). Around the onset of the 15M protests modularity collapses and displays an anti-correlated behavior with respect to nestedness. An exception to this is w = 3 days (panel d), in which the aggregated data in a single snapshot blurs the results of the community detection algorithm. A vertical green line has been placed on the time when nestedness and modularity bifurcate, and Pearson coefficients are reported for both sides of that line. A red horizontal line has been placed at $z = 1.96$, as a visual aid for statistical significance.

H. "Topic" null model

We have discussed in the beginning of this supplementary information distinct possibilities regarding the construction of presence-absence matrices that describe the set of interactions in our systems. We have also mentioned that different cutoffs to hashtags and/or users can be applied, and discussed the more reasonable way to proceed to study nestedness and modularity, which consists of either considering the most active users and their related set of hashtags, or the set of most active users plus most tweeted hashtags at a time. Also, on the side of statistical soundness, we have delved into different null model possibilities.

Now however, our concern focuses on the singularity of the results themselves. In particular, we want to test whether the modularity-nestedness crossover we have observed for particular topics is universal to *any* activity on Twitter (and in this sense uninteresting), or rather it is a specific mechanism underlying the formation of consensus around related information. Thus we explore here three additional possibilities for the w = 12h time-window on the UK dataset. In option (a) we select randomly and independently 512 users and 512 hashtags, and build the corresponding presence-absence matrix. Although the way in which nodes are selected can produce empty matrices corresponding to graphs with no links, this never happened in our dataset (all matrices have more than 20 non-empty cells). In model (b), 512 users are randomly selected and they determine the set of hashtags to consider. Model (c) is analogous but selecting randomly the 512 hashtags to be included, along with the set of users that tweeted them.

These three sets, (a), (b) and (c), can be considered as an additional category of null models that allow us to discern if the nested patterns previously observed are significant: for example, if set (a) showed high levels of z_λ we would not be able to conclude that the coordination phase observed in the 15M is relevant, as we would be finding nested patterns even for structures randomly filtered. A comparison between the three methods is displayed in Figure S14. Results include data from Figure 5 (bottom panel) in the main text. We observe that, when we consider independent users and hashtags at random –set (a)– nested patters do not show up and the bipartite network do not present any kind of organized structure. The exception is the region between the 3rd of February afternoon and the 4th of February, when the XLVII Super Bowl took place, probably due to the high relevance of this tournament (if it became global trending topic, even a randomly built network would show, to some extent, a nested structure). When users (hashtags) are randomly selected, but the set of hashtags (users) is closely related to them, the nestedness increase –sets (b) and (c)–, but this is a systematic shift rather than a differential change.

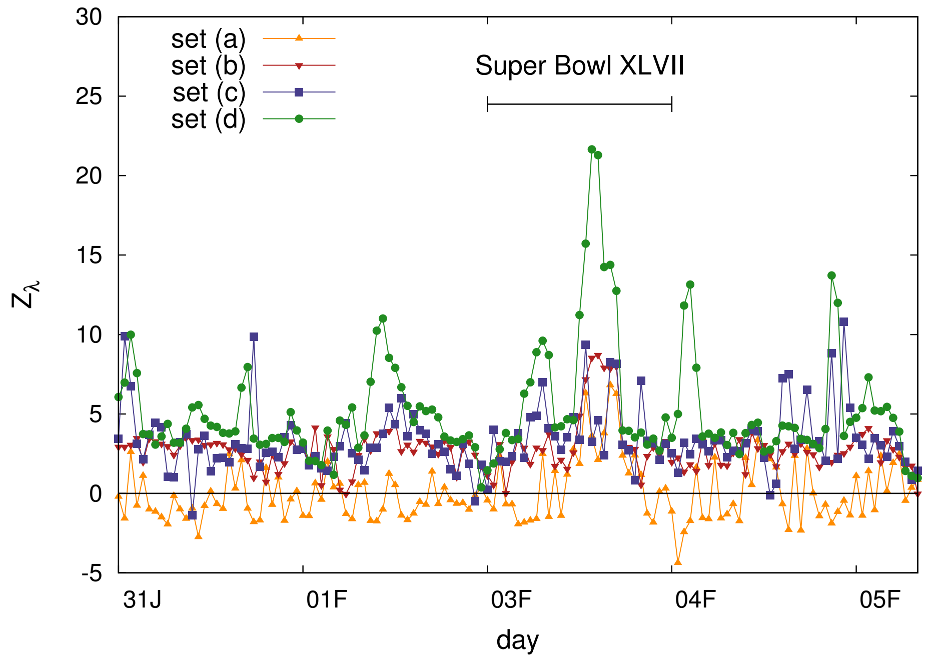

Figure S14: Some results for unfiltered Twitter traffic (2013). Set (a) corresponds to our UK dataset with 512 hashtags and 512 users randomly and independently selected. Such a random selection implies that the presence-absence matrix might be empty, although it never happened in this case. In set (b), 512 users have been randomly chosen determining the set of hashtags. Inversely, set (c) have been obtained by randomly filtering 512 hashtags their related users. Finally, set (4) comprises the 512 most active users and the 512 most used hashtags for comparison.

Appendix. Some selected hashtags

In Tables S3 and S4 we display some of the hashtags used in our dataset, along with the number of counts registered.

Hashtag	Counts	Hashtag	Counts
#acampadasol	189251	#acampadazgz	6033
#spanishrevolution	158487	#acamapadasol	5760
#nolesvotes	66329	#22m	5205
#15m	65962	#reflexion	4693
#nonosvamos	55245	#sinbanderas	4481
#democraciarealya	47463	#consensodeminimos	4348
#notenemosmiedo	32586	#italianrevolution	3981
#yeswecamp	31811	#estonosepara	3860
#acampadabcn	20069	#acampadaalicante	3593
#15mani	17986	#tomalacalle	3517
#acampadasevill1a	14356	#fb	3372
#globalcamp	13186	#europeanrevolution	3035
#acampadavalencia	13129	#acampadapamplona	2839
#estoesreflexion	11080	#worldrevolution	2777
#acampadagranada	9717	#democraciarealya	2766
#acampadamalaga	6808	#acampadapalma	2709

Table S3. Top 32 most-used hashtags in the 15M dataset.

Hashtag	Counts	Hashtag	Counts
#london	127082	#essex	38043
#ff	110972	#cbb	37741
#uk	80238	#lol	37646
#love	70618	#tired	37316
#jobs	70268	#legend	37225
#weather	66366	#summer	36739
#excited	64795	#cute	33648
#mufc	60176	#bgt	33586
#endomondo	55537	#coys	33380
#xfactor	51870	#wimbledon	32510
#lfc	47237	#nufc	32328
#stalbans	45117	#help	32118
#mtvhottest	43572	#amazing	30124
#happy	40086	#bored	30066
#loveit	40049	#bbuk	29960
#nowplaying	39798	#ukweather	29940

Table S4. Top 32 most-used hashtags in the UK dataset.

References

[1] Wu F, Huberman BA. Novelty and collective attention. Proc Natl Acad Sci USA 04(45):17599–17601 (2007).

[2] Crane R, Sornette D. Robust dynamic classes revealed by measuring the response function of a social system. Proc Natl Acad Sci USA 105(41):15649-15653 (2008).

[3] Leskovec J, Backstrom L, Kleinberg J. Meme-tracking and the dynamics of the news cycle. Proc 15th ACM SIGKDD pp. 497–506 (2009).

[4] Lehmann J, Gonçalves B, Ramasco JJ, Cattuto C. Dynamical classes of collective attention in twitter. Proc 21st Int Conf WWW pp. 251–260 (2012).

[5] Gonçalves B, Perra N, Vespignani A. Modeling users' activity on Twitter net- works: Validation of Dunbar's number. PloS One 6(8):e22656 (2011).

[6] Weng L, Flammini A, Vespignani A, Menczer F. Competition among memes in a world with limited attention. Sci Rep 2 (2012).

[7] Kleineberg, KK and Boguñá, M. Evolution of the digital society reveals balance between viral and mass media influence. Phys Rev X 4(3) 031046 (2014).

[8] Ribeiro, B and Faloutsos, C. Modeling Website Popularity Competition in the Attention-Activity Marketplace. Proc 8th ACM Intl Conf Web Search and Data Mining, 389–398 (2015).

[9] Cattuto C, Loreto V, Pietronero L. Semiotic dynamics and collaborative tagging. Proc Natl Acad Sci USA 104(5):1461–1464 (2007).

[10] Cattuto C, Barrat A, Baldassarri A, Schehr G, Loreto V. Collective dynamics of social annotation. Proc Natl Acad Sci USA 106(26):10511–10515 (2009).

[11] Gleeson JP, Ward JA, O'Sullivan KP, Lee WT. Competition-induced criticality in a model of meme popularity. Phys Rev Lett 112(4):048701 (2014).

[12] Bascompte J, Jordano P, Melián CJ, Olesen JM. The nested assembly of plant-animal mutualistic networks. Proc Natl Acad Sci USA 100(16):9383–9387 (2003).

[13] Bascompte J, Jordano P, Olesen JM. Asymmetric coevolutionary networks facilitate biodiversity maintenance. Science 312(5772):431–433 (2006).

[14] Bascompte J, Jordano P. Plant-animal mutualistic networks: the architecture of biodiversity. Ann Rev Ecol Evol System pp. 567–593 (2007).

[15] Saavedra S, Reed-Tsochas F, Uzzi B. A simple model of bipartite cooperation for ecological and organizational networks. Nature 457(7228):463–466 (2009).

[16] Bascompte J, Stouffer DB. The assembly and disassembly of ecological networks. Phil Trans Roy Soc B: Biol Sci 364(1524):1781–1787 (2009).

[17] Saavedra S, Stouffer DB, Uzzi B, Bascompte J. Strong contributors to network persistence are the most vulnerable to extinction. Nature 478(7368):233–235 (2011).

[18] Jonhson S, Domínguez-García V, Muñoz MA. Factors determining nestedness in complex networks. PloS One 8(9):e74025 (2013).

[19] Rohr RP, Saavedra S, Bascompte J. On the structural stability of mutualistic systems. Science 345(6195):1253497 (2014).

[20] Thébault E, Fontaine C. Stability of ecological communities and the architecture of mutualistic and trophic networks. Science 329(5993):853–856 (2010).

[21] Stouffer DB, Bascompte J. Compartmentalization increases food-web persistence. Proc Natl Acad Sci USA 108(9):3648–3652 (2011).

[22] Guimerà, R and Stouffer, DB and Sales-Pardo, M and Leicht, EA and Newman, MEJ and Amaral, LAN. Origin of compartmentalization in food webs. Ecology 91(10):2941–2951 (2010).

[23] Borge-Holthoefer J *et al*. Structural and dynamical patterns on online social networks: the Spanish May 15th movement as a case study. PLoS One 6(8):e23883 (2011).

[24] González-Bailón S, Borge-Holthoefer J, Rivero A, Moreno Y. The dynamics of protest recruitment through an online network. Sci Rep 1:197 (2011).

[25] Fortunato S. Community detection in graphs. Phys Rep 486(3-5):75–174 (2010).

[26] Barber MJ (2007) Modularity and community detection in bipartite networks. Phys Rev E 76(6):066102.

[27] Bell FK, Cvetković D, Rowlinson P, Simić SK. Graphs for which the least eigenvalue is minimal, I. Lin Alg App 429(1):234–241 (2008).

[28] Bell FK, Cvetković D, Rowlinson P, Simić SK. Graphs for which the least eigenvalue is minimal, II. Lin Alg App 429(8):2168–2179 (2008).

[29] Staniczenko PP, Kopp JC, Allesina S. The ghost of nestedness in ecological networks. Nat Comm 4:1391 (2013).

[30] Almeida-Neto M, Guimarães P, Guimarães PR, Loyola RD, Ulrich W. A consistent metric for nestedness analysis in ecological systems: reconciling concept and measurement. Oikos 117(8):1227–1239 (2008).

[31] May RM, Levin SA, Sugihara G. Complex systems: Ecology for bankers. Nature 451(7181):893–895 (2008).

[32] Kamilar JM, Atkinson QD. Cultural assemblages show nested structure in humans and chimpanzees but not orangutans. Proc Natl Acad Sci USA 111(1):111–115. 32 (2014).

[33] Alarcón R, Waser NM, Ollerton J. Year-to-year variation in the topology of a plant–pollinator interaction network. Oikos 117(12):1796–1807 (2008).

[34] Olesen JM, Bascompte J, Elberling H, Jordano P. Temporal dynamics in a pollination network. Ecology 89(6):1573–1582 (2008).

[35] Díaz-Castelazo C *et al*. Changes of a mutualistic network over time: reanalysis over a 10-year period. Ecology 91(3):793–801 (2010).

[36] Bastolla U *et al*. The architecture of mutualistic networks minimizes competition and increases biodiversity. Nature 458(7241):1018–1020 (2009).

[37] Borgatti S, Everett M. Models of core-periphery structures. Soc Net 21:375–395 (1999).

[38] Della Rossa F, Dercole F, Piccardi C. Profiling core-periphery network structure by random walkers. Sci Rep 3 (2013).

[39] Domínguez-García V, Muñoz MA. Ranking species in mutualistic networks. Sci Rep 5 (2015).

[40] Borge-Holthoefer J, Perra N, González-Bailón S, Gonçalves B, Arenas A, Moreno Y, Vespignani A. The dynamic of information-driven coordination phenomena: a transfer entropy analysis. Sci Adv 2(4) e1501158 (2016).

[41] Olesen JM, Bascompte J, Dupont YL, Jordano P. The modularity of pollination networks. Proc Natl Acad Sci USA 104(50):19891–19896 (2007).

[42] Fortuna MA *et al*. Nestedness versus modularity in ecological networks: two sides of the same coin? J Anim Ecol 79(4):811–817 (2010).

[43] Flores CO, Meyer JR, Valverde S, Farr L, Weitz JS. Statistical structure of host–phage interactions. Proc Natl Acad Sci USA 108(28):E288–E297 (2011).

[44] Guimerà R, Sales-Pardo M, Amaral LAN. Modularity from fluctuations in random graphs and complex networks. Phys Rev E 70(2):025101 (2004).

[45] Atmar W, Patterson BD. The measure of order and disorder in the distribution of species in fragmented habitat. Oecologia 96(3):373–382 (1993).

[46] Darlington PJ. Zoogeography: the geographical distribution of animals (1957).

[47] Daubenmire R. Floristic plant geography of Eastern Washington and Northern Idaho. J Biogeo (1975).

[48] Guimerà R, Sales-Pardo M, Amaral LAN. Module identification in bipartite and directed networks. Phys Rev E 76(3):036102 (2007).

[49] Hultén E. Outline of the history of arctic and boreal biota during the Quaternary period. (Thule, Stockholm) (1937).

[50] Marquitti FM, Guimaraes PR, Pires MM, Bittencourt LF. Modular: software for the autonomous computation of modularity in large network sets. Ecography 37(3):221–224 (2014).

[51] Newman MEJ. Fast algorithm for detecting community structure in networks. Phys Rev E 69 (2004).

[52] Patterson BD, Atmar W. Nested subsets and the structure of insular mammalian faunas and archipelagos. Biol J Linn Soc 28(1- 2):65–82 (1986).

[53] Petanidou T, Kallimanis AS, Tzanopoulos J, Sgardelis SP, Pantis JD. Long-term observation of a pollination network: fluctuation in species and interactions, relative invariance of network structure and implications for estimates of specialization. Ecol Lett 11(6):564–575 (2008).

[54] Saavedra S, Rohr RP, Dakos V, Bascompte J. Estimating the tolerance of species to the effects of global environmental change. Nat Comm 4:1038 (2013).

[55] Thompson, J. N. The geographic mosaic of coevolution. (University of Chicago Press) (2005).
